# 2-[4-Acetyl-5-(biphenyl-4-yl)-4,5-dihydro-1,3,4-oxadiazol-2-yl]phenyl acetate

**DOI:** 10.1107/S1600536810009621

**Published:** 2010-03-20

**Authors:** Wagee A. Yehye, Azhar Ariffin, Noorsaadah Abdul Rahman, Seik Weng Ng

**Affiliations:** a Department of Chemistry, University of Malaya, 50603 Kuala Lumpur, Malaysia

## Abstract

In the title mol­ecule, C_24_H_20_N_2_O_4_, the five-membered oxadiazole ring is nearly planar (r.m.s. deviation = 0.053 Å) and the phenyl ring of the biphenyl unit attached to it forms a dihedral angle of 73.2 (1)°; the other phenyl ring is close to coplanar with the oxadiazole ring [dihedral angle = 6.2 (2)°].

## Related literature

For the crystal structures of other 2,3-dihydro-1,3,4-oxa­diazo­les, see: Jin *et al.* (2006 ▶); Somogyi *et al.* (1992 ▶); Song *et al.* (2006 ▶); He & Zhu (2008 ▶).
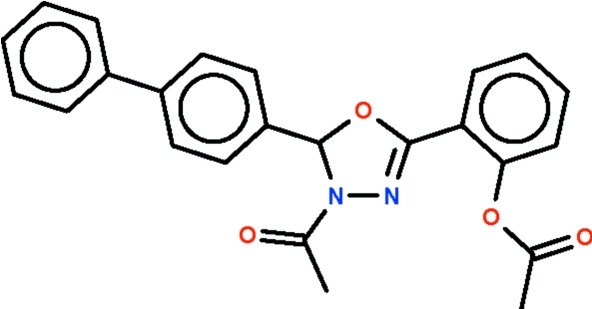


## Experimental

### 

#### Crystal data

C_24_H_20_N_2_O_4_
*M* *_r_* = 400.42
Orthorhombic, 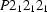

*a* = 8.6750 (11) Å
*b* = 12.0279 (14) Å
*c* = 19.221 (2) Å
*V* = 2005.6 (4) Å^3^
*Z* = 4
Mo *K*α radiation
μ = 0.09 mm^−1^
*T* = 293 K
0.30 × 0.20 × 0.10 mm

#### Data collection

Bruker SMART APEX diffractometer
16090 measured reflections
2029 independent reflections
1534 reflections with *I* > 2σ(*I*)
*R*_int_ = 0.056

#### Refinement

*R*[*F* ^2^ > 2σ(*F* ^2^)] = 0.035
*wR*(*F* ^2^) = 0.100
*S* = 1.00
2029 reflections
274 parameters
H-atom parameters constrained
Δρ_max_ = 0.11 e Å^−3^
Δρ_min_ = −0.13 e Å^−3^

### 

Data collection: *APEX2* (Bruker, 2009 ▶); cell refinement: *SAINT* (Bruker, 2009 ▶); data reduction: *SAINT*; program(s) used to solve structure: *SHELXS97* (Sheldrick, 2008 ▶); program(s) used to refine structure: *SHELXL97* (Sheldrick, 2008 ▶); molecular graphics: *X-SEED* (Barbour, 2001 ▶); software used to prepare material for publication: *publCIF* (Westrip, 2010 ▶).

## Supplementary Material

Crystal structure: contains datablocks global, I. DOI: 10.1107/S1600536810009621/pv2264sup1.cif

Structure factors: contains datablocks I. DOI: 10.1107/S1600536810009621/pv2264Isup2.hkl

Additional supplementary materials:  crystallographic information; 3D view; checkCIF report

## Figures and Tables

**Fig. 1.**
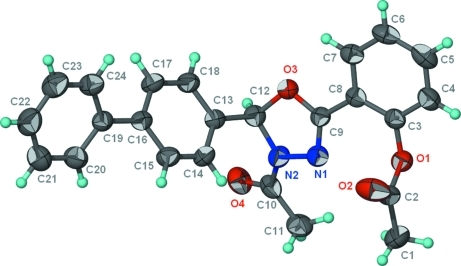
Thermal ellipsoid plot (Barbour, 2001) of the title compound; ellipsoids are drawn at the 50% probability level and H atoms are of arbitrary radiuii.

## supplementary crystallographic information

### Comment

The Schiff base reactant, *N*'-(4-phenylbenzylidene)-2-hydroxybenzohydrazide, features an –C(=O)–NH–N=CH linkage betwen the two aromatic systems that can be induced to form an oxadiazole. In this study, the oxadiazole is indeed formed when cyclized in acetic anhyride. The nitrogen atom in the 3-position of the ring has also been acetylated; interestingly, the hydroxy group also undergoes acetylation to yield a bis-acetylated product, the title compound. The crystal structure of the title compound is reported in this article (Fig. 1).

### Experimental

The Schiff base, *N*'-(4-phenylbenzylidene)-2-hydroxybenzohydrazide, was synthesized by condensing 4-phenylbenzaldehyde with 2-hydrobenzhydrazide. The compound (0.5 g, 1.58 mmol) was heated in acetic anhydride (5 ml) for 2 h. The solution was cooled and then poured into crushed ice. The solid that separated solid was collected and recrystallized from methanol to give the title compound as well-formed prisms.

### Refinement

The H-atoms were placed in calculated positions (C—H 0.93–98 Å) and were included in the refinement in the riding model approximation, with *U_iso_*(H) set to 1.2–1.5*U_eq_*(C). Due to insufficient anomalous dispersion effects, an absolute configuration could not be established. Therefore, 1495 Friedel pairs were merged.

### Crystal data

**Table:**

C_24_H_20_N_2_O_4_	*F*(000) = 840
*M**_r_* = 400.42	*D*_x_ = 1.326 Mg m^−^^3^
Orthorhombic, *P*2_1_2_1_2_1_	Mo *K*α radiation, λ = 0.71073 Å
Hall symbol: P 2ac 2ab	Cell parameters from 2292 reflections
*a* = 8.6750 (11) Å	θ = 2.6–19.9°
*b* = 12.0279 (14) Å	µ = 0.09 mm^−^^1^
*c* = 19.221 (2) Å	*T* = 293 K
*V* = 2005.6 (4) Å^3^	Prism, colorless
*Z* = 4	0.30 × 0.20 × 0.10 mm

### Data collection

**Table:**

Bruker SMART APEX diffractometer	1534 reflections with *I* > 2σ(*I*)
Radiation source: fine-focus sealed tube	*R*_int_ = 0.056
graphite	θ_max_ = 25.0°, θ_min_ = 2.0°
ω scans	*h* = −10→10
16090 measured reflections	*k* = −14→14
2029 independent reflections	*l* = −22→22

### Refinement

**Table:**

Refinement on *F*^2^	Secondary atom site location: difference Fourier map
Least-squares matrix: full	Hydrogen site location: inferred from neighbouring sites
*R*[*F*^2^ > 2σ(*F*^2^)] = 0.035	H-atom parameters constrained
*wR*(*F*^2^) = 0.100	*w* = 1/[σ^2^(*F*_o_^2^) + (0.0598*P*)^2^ + 0.076*P*] where *P* = (*F*_o_^2^ + 2*F*_c_^2^)/3
*S* = 1.00	(Δ/σ)_max_ = 0.001
2029 reflections	Δρ_max_ = 0.11 e Å^−^^3^
274 parameters	Δρ_min_ = −0.13 e Å^−^^3^
0 restraints	Extinction correction: *SHELXL97* (Sheldrick, 2008), Fc^*^=kFc[1+0.001xFc^2^λ^3^/sin(2θ)]^-1/4^
Primary atom site location: structure-invariant direct methods	Extinction coefficient: 0.0082 (18)

### Fractional atomic coordinates and isotropic or equivalent isotropic displacement parameters (Å^2^)

**Table:**

	*x*	*y*	*z*	*U*_iso_*/*U*_eq_	
O1	−0.0208 (3)	1.13163 (16)	0.16056 (10)	0.0571 (6)	
O2	−0.1684 (3)	1.0247 (2)	0.22783 (14)	0.0894 (9)	
O3	0.3885 (2)	0.96800 (17)	0.22120 (10)	0.0540 (5)	
O4	0.4949 (3)	1.19201 (19)	0.37185 (12)	0.0706 (7)	
N1	0.2135 (3)	1.09315 (19)	0.25521 (13)	0.0513 (6)	
N2	0.3385 (3)	1.09851 (19)	0.30133 (13)	0.0537 (7)	
C1	−0.1667 (5)	1.2206 (3)	0.2462 (2)	0.0806 (11)	
H1A	−0.2221	1.2060	0.2884	0.121*	
H1B	−0.2307	1.2627	0.2151	0.121*	
H1C	−0.0751	1.2622	0.2566	0.121*	
C2	−0.1239 (4)	1.1146 (3)	0.21312 (17)	0.0589 (8)	
C3	0.0343 (4)	1.0375 (2)	0.12550 (15)	0.0488 (7)	
C4	−0.0388 (4)	1.0074 (3)	0.06492 (16)	0.0618 (9)	
H4	−0.1250	1.0465	0.0497	0.074*	
C5	0.0166 (5)	0.9191 (3)	0.02688 (17)	0.0693 (10)	
H5	−0.0325	0.8978	−0.0140	0.083*	
C6	0.1450 (4)	0.8622 (3)	0.04945 (17)	0.0631 (9)	
H6	0.1821	0.8024	0.0238	0.076*	
C7	0.2189 (4)	0.8933 (2)	0.10977 (15)	0.0534 (8)	
H7	0.3055	0.8541	0.1244	0.064*	
C8	0.1655 (3)	0.9829 (2)	0.14935 (14)	0.0442 (7)	
C9	0.2499 (3)	1.0175 (2)	0.21116 (15)	0.0451 (7)	
C10	0.3703 (4)	1.1884 (2)	0.34137 (16)	0.0550 (8)	
C11	0.2494 (5)	1.2753 (3)	0.34728 (19)	0.0733 (10)	
H11A	0.2971	1.3461	0.3556	0.110*	
H11B	0.1818	1.2576	0.3852	0.110*	
H11C	0.1913	1.2784	0.3048	0.110*	
C12	0.4469 (4)	1.0077 (2)	0.28773 (15)	0.0518 (7)	
H12	0.5521	1.0363	0.2827	0.062*	
C13	0.4419 (3)	0.9151 (2)	0.34025 (15)	0.0443 (7)	
C14	0.3338 (4)	0.9088 (3)	0.39249 (16)	0.0534 (8)	
H14	0.2617	0.9654	0.3975	0.064*	
C15	0.3307 (4)	0.8198 (3)	0.43733 (16)	0.0562 (8)	
H15	0.2570	0.8177	0.4724	0.067*	
C16	0.4350 (3)	0.7333 (2)	0.43137 (15)	0.0463 (7)	
C17	0.5437 (4)	0.7399 (3)	0.37835 (17)	0.0568 (8)	
H17	0.6146	0.6826	0.3728	0.068*	
C18	0.5483 (4)	0.8292 (2)	0.33416 (16)	0.0557 (8)	
H18	0.6234	0.8325	0.2997	0.067*	
C19	0.4308 (3)	0.6360 (2)	0.47964 (15)	0.0486 (7)	
C20	0.4008 (4)	0.6501 (3)	0.54999 (17)	0.0651 (9)	
H20	0.3819	0.7210	0.5673	0.078*	
C21	0.3985 (5)	0.5602 (3)	0.5946 (2)	0.0803 (12)	
H21	0.3795	0.5708	0.6418	0.096*	
C22	0.4242 (5)	0.4550 (3)	0.5693 (2)	0.0804 (12)	
H22	0.4192	0.3942	0.5991	0.096*	
C23	0.4573 (5)	0.4395 (3)	0.5002 (2)	0.0758 (11)	
H23	0.4778	0.3685	0.4834	0.091*	
C24	0.4601 (4)	0.5292 (3)	0.45577 (18)	0.0610 (8)	
H24	0.4819	0.5181	0.4089	0.073*	

### Atomic displacement parameters (Å^2^)

**Table:**

	*U*^11^	*U*^22^	*U*^33^	*U*^12^	*U*^13^	*U*^23^
O1	0.0657 (14)	0.0503 (12)	0.0552 (12)	0.0078 (10)	0.0035 (11)	0.0030 (10)
O2	0.103 (2)	0.0775 (17)	0.0877 (18)	−0.0177 (17)	0.0411 (16)	−0.0123 (16)
O3	0.0576 (13)	0.0552 (12)	0.0492 (11)	0.0090 (10)	0.0011 (10)	−0.0022 (10)
O4	0.0828 (18)	0.0671 (15)	0.0619 (14)	−0.0174 (13)	−0.0126 (14)	−0.0039 (12)
N1	0.0569 (16)	0.0432 (14)	0.0538 (15)	−0.0015 (12)	−0.0060 (13)	−0.0069 (13)
N2	0.0579 (16)	0.0417 (13)	0.0614 (15)	0.0022 (12)	−0.0096 (14)	−0.0074 (13)
C1	0.080 (3)	0.080 (3)	0.082 (3)	0.029 (2)	−0.003 (2)	−0.010 (2)
C2	0.0554 (19)	0.067 (2)	0.0539 (19)	0.0090 (17)	0.0042 (17)	−0.0071 (18)
C3	0.0570 (18)	0.0457 (16)	0.0438 (16)	−0.0027 (15)	0.0071 (15)	0.0039 (14)
C4	0.0587 (19)	0.077 (2)	0.0496 (19)	−0.0057 (19)	0.0016 (17)	0.0002 (17)
C5	0.072 (2)	0.088 (3)	0.0487 (19)	−0.020 (2)	0.0041 (18)	−0.0086 (19)
C6	0.074 (2)	0.061 (2)	0.055 (2)	−0.0131 (19)	0.0136 (19)	−0.0187 (17)
C7	0.0576 (19)	0.0507 (18)	0.0518 (18)	−0.0051 (16)	0.0093 (16)	−0.0033 (15)
C8	0.0528 (16)	0.0371 (14)	0.0429 (16)	−0.0075 (13)	0.0058 (14)	0.0042 (13)
C9	0.0511 (16)	0.0368 (15)	0.0474 (17)	0.0009 (13)	0.0030 (14)	0.0021 (14)
C10	0.077 (2)	0.0436 (17)	0.0445 (17)	−0.0122 (17)	−0.0019 (18)	0.0036 (14)
C11	0.101 (3)	0.0514 (19)	0.067 (2)	0.003 (2)	0.000 (2)	−0.0150 (18)
C12	0.0507 (16)	0.0515 (18)	0.0532 (18)	−0.0041 (15)	−0.0030 (15)	−0.0049 (15)
C13	0.0403 (15)	0.0447 (15)	0.0480 (16)	−0.0029 (13)	−0.0015 (14)	−0.0043 (14)
C14	0.0498 (18)	0.0476 (18)	0.063 (2)	0.0096 (15)	0.0021 (16)	0.0006 (16)
C15	0.0501 (17)	0.0585 (19)	0.0600 (19)	0.0033 (16)	0.0147 (16)	0.0026 (17)
C16	0.0420 (16)	0.0461 (16)	0.0507 (17)	−0.0021 (14)	0.0002 (15)	−0.0069 (14)
C17	0.0542 (18)	0.0513 (18)	0.0651 (19)	0.0118 (16)	0.0066 (17)	−0.0032 (16)
C18	0.0538 (18)	0.0588 (18)	0.0546 (18)	0.0054 (16)	0.0115 (16)	0.0020 (16)
C19	0.0398 (16)	0.0527 (18)	0.0534 (18)	0.0000 (14)	−0.0006 (14)	−0.0025 (15)
C20	0.074 (2)	0.061 (2)	0.059 (2)	0.0033 (18)	0.0058 (19)	−0.0022 (18)
C21	0.092 (3)	0.087 (3)	0.062 (2)	0.006 (2)	0.008 (2)	0.012 (2)
C22	0.081 (3)	0.076 (3)	0.085 (3)	0.007 (2)	0.012 (2)	0.030 (2)
C23	0.076 (2)	0.057 (2)	0.094 (3)	0.0040 (19)	0.012 (2)	0.012 (2)
C24	0.062 (2)	0.0540 (19)	0.067 (2)	0.0000 (18)	0.0093 (18)	−0.0013 (17)

### Geometric parameters (Å, °)

**Table:**

O1—C2	1.365 (4)	C11—H11B	0.9600
O1—C3	1.401 (3)	C11—H11C	0.9600
O2—C2	1.183 (4)	C12—C13	1.503 (4)
O3—C9	1.356 (4)	C12—H12	0.9800
O3—C12	1.456 (3)	C13—C14	1.376 (4)
O4—C10	1.230 (4)	C13—C18	1.390 (4)
N1—C9	1.283 (3)	C14—C15	1.374 (4)
N1—N2	1.402 (3)	C14—H14	0.9300
N2—C10	1.355 (4)	C15—C16	1.384 (4)
N2—C12	1.465 (4)	C15—H15	0.9300
C1—C2	1.472 (4)	C16—C17	1.391 (4)
C1—H1A	0.9600	C16—C19	1.494 (4)
C1—H1B	0.9600	C17—C18	1.370 (4)
C1—H1C	0.9600	C17—H17	0.9300
C3—C4	1.374 (4)	C18—H18	0.9300
C3—C8	1.392 (4)	C19—C20	1.387 (4)
C4—C5	1.377 (5)	C19—C24	1.387 (4)
C4—H4	0.9300	C20—C21	1.381 (5)
C5—C6	1.377 (5)	C20—H20	0.9300
C5—H5	0.9300	C21—C22	1.374 (5)
C6—C7	1.376 (4)	C21—H21	0.9300
C6—H6	0.9300	C22—C23	1.371 (5)
C7—C8	1.398 (4)	C22—H22	0.9300
C7—H7	0.9300	C23—C24	1.377 (5)
C8—C9	1.456 (4)	C23—H23	0.9300
C10—C11	1.485 (5)	C24—H24	0.9300
C11—H11A	0.9600		
			
C2—O1—C3	117.3 (2)	H11B—C11—H11C	109.5
C9—O3—C12	106.9 (2)	O3—C12—N2	100.2 (2)
C9—N1—N2	105.1 (2)	O3—C12—C13	109.7 (2)
C10—N2—N1	123.6 (3)	N2—C12—C13	114.4 (2)
C10—N2—C12	124.4 (3)	O3—C12—H12	110.7
N1—N2—C12	110.4 (2)	N2—C12—H12	110.7
C2—C1—H1A	109.5	C13—C12—H12	110.7
C2—C1—H1B	109.5	C14—C13—C18	118.2 (3)
H1A—C1—H1B	109.5	C14—C13—C12	123.4 (3)
C2—C1—H1C	109.5	C18—C13—C12	118.3 (3)
H1A—C1—H1C	109.5	C15—C14—C13	120.9 (3)
H1B—C1—H1C	109.5	C15—C14—H14	119.5
O2—C2—O1	121.9 (3)	C13—C14—H14	119.5
O2—C2—C1	127.3 (3)	C14—C15—C16	121.4 (3)
O1—C2—C1	110.8 (3)	C14—C15—H15	119.3
C4—C3—C8	122.1 (3)	C16—C15—H15	119.3
C4—C3—O1	117.6 (3)	C15—C16—C17	117.4 (3)
C8—C3—O1	120.1 (3)	C15—C16—C19	121.5 (3)
C3—C4—C5	119.5 (3)	C17—C16—C19	121.1 (3)
C3—C4—H4	120.3	C18—C17—C16	121.3 (3)
C5—C4—H4	120.3	C18—C17—H17	119.4
C4—C5—C6	119.9 (3)	C16—C17—H17	119.4
C4—C5—H5	120.1	C17—C18—C13	120.7 (3)
C6—C5—H5	120.1	C17—C18—H18	119.6
C7—C6—C5	120.5 (3)	C13—C18—H18	119.6
C7—C6—H6	119.8	C20—C19—C24	118.0 (3)
C5—C6—H6	119.8	C20—C19—C16	120.9 (3)
C6—C7—C8	120.9 (3)	C24—C19—C16	121.0 (3)
C6—C7—H7	119.6	C21—C20—C19	120.8 (3)
C8—C7—H7	119.6	C21—C20—H20	119.6
C3—C8—C7	117.1 (3)	C19—C20—H20	119.6
C3—C8—C9	123.0 (3)	C22—C21—C20	119.9 (3)
C7—C8—C9	119.9 (3)	C22—C21—H21	120.1
N1—C9—O3	115.8 (3)	C20—C21—H21	120.1
N1—C9—C8	128.2 (3)	C23—C22—C21	120.2 (3)
O3—C9—C8	115.9 (2)	C23—C22—H22	119.9
O4—C10—N2	118.5 (3)	C21—C22—H22	119.9
O4—C10—C11	124.0 (3)	C22—C23—C24	119.8 (3)
N2—C10—C11	117.5 (3)	C22—C23—H23	120.1
C10—C11—H11A	109.5	C24—C23—H23	120.1
C10—C11—H11B	109.5	C23—C24—C19	121.1 (3)
H11A—C11—H11B	109.5	C23—C24—H24	119.4
C10—C11—H11C	109.5	C19—C24—H24	119.4
H11A—C11—H11C	109.5		
			
C9—N1—N2—C10	158.3 (3)	C10—N2—C12—O3	−154.2 (3)
C9—N1—N2—C12	−8.2 (3)	N1—N2—C12—O3	12.1 (3)
C3—O1—C2—O2	−1.7 (4)	C10—N2—C12—C13	88.5 (3)
C3—O1—C2—C1	178.1 (3)	N1—N2—C12—C13	−105.2 (3)
C2—O1—C3—C4	95.3 (3)	O3—C12—C13—C14	−104.7 (3)
C2—O1—C3—C8	−89.4 (3)	N2—C12—C13—C14	7.0 (4)
C8—C3—C4—C5	1.4 (5)	O3—C12—C13—C18	72.7 (3)
O1—C3—C4—C5	176.6 (3)	N2—C12—C13—C18	−175.6 (3)
C3—C4—C5—C6	−0.5 (5)	C18—C13—C14—C15	−0.1 (4)
C4—C5—C6—C7	−0.2 (5)	C12—C13—C14—C15	177.3 (3)
C5—C6—C7—C8	0.0 (5)	C13—C14—C15—C16	−0.5 (5)
C4—C3—C8—C7	−1.6 (4)	C14—C15—C16—C17	0.3 (4)
O1—C3—C8—C7	−176.6 (2)	C14—C15—C16—C19	−179.4 (3)
C4—C3—C8—C9	176.3 (3)	C15—C16—C17—C18	0.6 (4)
O1—C3—C8—C9	1.3 (4)	C19—C16—C17—C18	−179.7 (3)
C6—C7—C8—C3	0.9 (4)	C16—C17—C18—C13	−1.3 (5)
C6—C7—C8—C9	−177.1 (3)	C14—C13—C18—C17	1.0 (4)
N2—N1—C9—O3	0.1 (3)	C12—C13—C18—C17	−176.5 (3)
N2—N1—C9—C8	−175.7 (3)	C15—C16—C19—C20	−39.6 (4)
C12—O3—C9—N1	7.9 (3)	C17—C16—C19—C20	140.8 (3)
C12—O3—C9—C8	−175.8 (2)	C15—C16—C19—C24	141.8 (3)
C3—C8—C9—N1	6.3 (4)	C17—C16—C19—C24	−37.8 (4)
C7—C8—C9—N1	−175.9 (3)	C24—C19—C20—C21	−0.7 (5)
C3—C8—C9—O3	−169.5 (2)	C16—C19—C20—C21	−179.3 (3)
C7—C8—C9—O3	8.3 (4)	C19—C20—C21—C22	−0.8 (6)
N1—N2—C10—O4	−168.8 (3)	C20—C21—C22—C23	2.2 (7)
C12—N2—C10—O4	−4.3 (4)	C21—C22—C23—C24	−2.0 (6)
N1—N2—C10—C11	12.9 (4)	C22—C23—C24—C19	0.4 (6)
C12—N2—C10—C11	177.4 (3)	C20—C19—C24—C23	0.9 (5)
C9—O3—C12—N2	−11.5 (3)	C16—C19—C24—C23	179.6 (3)
C9—O3—C12—C13	109.3 (3)		
